# Successful reconstruction of the posterior cruciate ligament: assessment of posterior cruciate ligament footprints using an objective coordinate system

**DOI:** 10.1007/s00276-020-02520-9

**Published:** 2020-06-17

**Authors:** Ines Vielgut, Andreas Weiglein, Stefan M. Biber, Manuel Dreu, Andreas Leithner, Goria Hohenberger, Patrick Sadoghi

**Affiliations:** 1 Department of Orthopaedics and Trauma, Medical University of Graz, Auenbruggerplatz 5, 8036 Graz, Austria; 2 Institute of Anatomy, Medical University of Graz, Harrachgasse 21/1 HG, 8010 Graz, Austria

**Keywords:** Anatomical study, PCL, Reconstruction, Tunnel positioning

## Abstract

**Introduction:**

Anatomic cruciate ligament reconstruction is known to be correlated with better clinical results. The aim of the study was to provide a simple method to enable anatomic results in the setting of PCL reconstruction. We, therefore, assessed the tibial and femoral insertion site of the posterior cruciate ligament (PCL) by the use of an objective coordinate system in an anatomical study. We also sought to show reproducibility of these measurements using intra- and inter-observer coefficients.

**Materials and methods:**

We studied 64 knees, previously preserved according to Thiel’s technique. After proper preparation of the articular surfaces of both the tibiae and femora, photographs were taken according to a standardized protocol. PCL footprints were measured by the use of a coordinate system twice by two examiners. We evaluated these measurements by use of the Cohen’s kappa inter- and intra-observer coefficient for two observers.

**Results:**

Tibial and femoral measurements of PCL footprints were generated with highly comparable inter- (*k* = 0.970) and intra-observer (*k* = 0.992) coefficients and may, therefore, be considered as highly reproducible.

**Conclusion:**

Our findings confirmed the reproducibility of defining PCL footprints using a coordinate system and may contribute to planning intraoperative graft-placement to ensure optimal conditions in the upcoming techniques for PCL reconstruction.

## Introduction

Injuries to the posterior cruciate ligament are reported to comprise approximately 3% of all knee ligament injuries in the general population [6, 9, 14, 16, 20, 24, 29]. PCL injuries rarely exist in isolation, and typically occur concurrently with other knee injuries, including ACL, medial collateral ligament (MCL), or posterolateral corner (PLC) injuries [11].

Anatomic single-bundle reconstruction of the PCL, using arthroscopic and radiographic reference points have focused on current literature and more and more replaces the historical “isometric” reconstruction, where it was assumed that the femoral insertion point of the PCL maintains a fixed distance from a single point on tibia (tibial insertion site of the PCL) during range of motion (ROM). Reconstruction techniques which were based on this theory of an “isometric” PCL, have been reported to result in initial joint over-constraint and increased laxity over time, as it is accepted that the length of the ligament varies, depending on the tension on the ligament during ROM [10, 11].

Studies have described PCL reconstructions where the femoral and tibial residuals of the PCL are preserved to anatomically reinsert the PCL replacement graft [1, 3, 4, 12, 13, 16, 17]. This technique is hypothesized to enhance healing and transplant survival due to increased vascular ingrowth [21, 28]. The most commonly reported negative impacts after PCL reconstruction are residual posterior laxity, flexion loss, and osteoarthritis as a long-term complication [24, 30].

Therefore, understanding PCL anatomy including ligament footprints is mandatory to achieve optimal results in PCL reconstruction [4, 15, 19].

The purpose of this study was to precisely assess tibial and femoral insertion of the PCL using an objective coordinate system in an anatomical study on donated bodies to science.

Equally, we also sought to demonstrate the reproducibility of these measurements using intra- and inter-observer coefficients.

## Methods

Ethical approval was obtained from the Ethics Committee of the Medical University of Graz. All bodies were donated to science, provided to the Department of Anatomy of the Medical University of Graz, under approval of the Anatomical Donation Program of the University of Graz, and the Austrian law for donations.

We studied 64 knees which were previously preserved using Thiel’s technique [26]. The joints were taken from 30 male and 34 female bodies, with a mean age of 75 years at death (range, 41–101 years). The bodies had a mean height of 167.7 cm (range, 150–182 cm) and a mean weight of 67.3 kg (range, 46–115 kg).

After the PCL was identified, both the cruciate ligaments, as well as the medial and lateral collateral ligaments were transected. Subsequently, the center of the tibial and the femoral footprint was visually identified and marked with a pen.

**Fig. 1:**
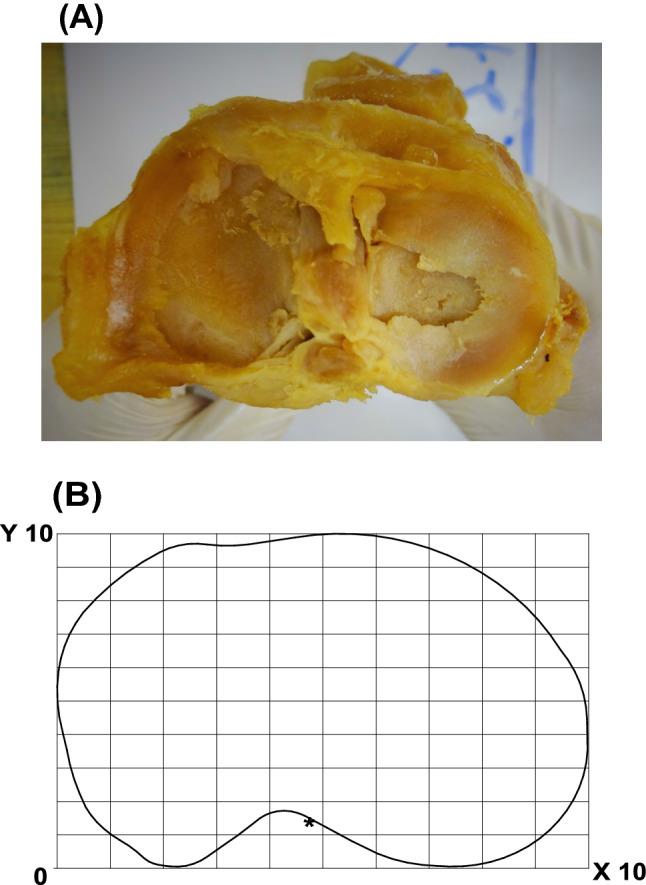
**a** This picture shows the cranio-caudal view on the tibial plateau. All tibiae were photographed in this way to assess the tibial PCL insertion site by the use of an overlaid standardised coordinate system. **b** This sketch illustrates the standardised coordinate system overlaid onto a picture of a tibial plateau from a cranio-caudal view

In case of a macroscopic double-bundle PCL, we chose the midpoint of these two bundles for assessment. After this, we photographed the tibiae according to criteria from a previously described protocol [19], and used an analogue setting to describe anatomical footprints of the PCL (Fig. 1a).

**Fig. 2:**
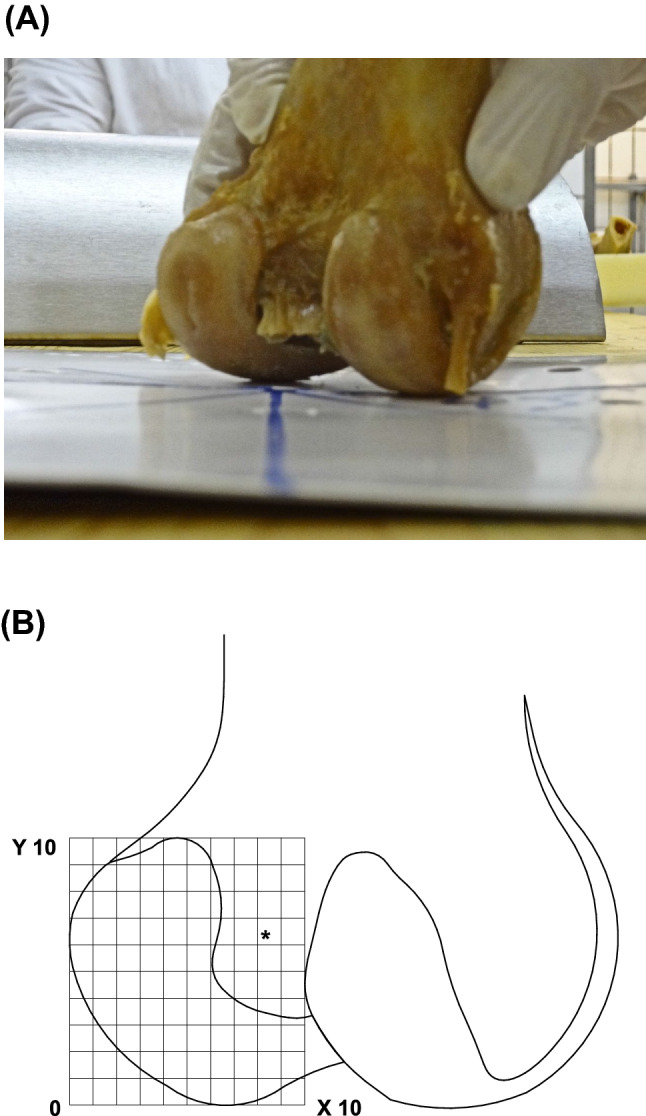
**a** This picture illustrates the standardized photograph of a 45° inwards rotated femur. **b** This sketch illustrates the standardised coordinate system which was overlaid onto a standardized photograph of a 45° inwards rotated femur

To optimize presentation of the intercondylar notch and the femoral PCL footprint, we rotated the knee from a strict posterior–anterior femoral view, in a 45° external rotated position. This rotation was verified using a conventional graphometer circle (Fig. 2a).

Photographs were taken by both observers, and standardized photographs were printed to assess tibial and femoral footprints using a coordinate system, which was manually drawn.

### The coordinate system

Both the tibial footprint of the PCL and coordinate values were drawn and measured on a strict cranio-caudal view. The coordinate system was oriented on the following anatomical landmarks: the zero values of the *X*- and *Y*-axes represented the posteromedial corner of a rectangle drawn around the photograph of each tibial plateau. The length and width of the circumscribing rectangle was divided into ten equally large sections (Fig. 1b).

For the femur, the *X*-coordinate ranged from the cranial beginning of the condyle to its cranial end. The *Y*-coordinate ranged from the articulating femoral edge to the outer (lateral) edge of the lateral condyle (Fig. 2b).

Finally, the previously marked footprints of the PCL were defined using the sectioned coordinate system.

All preparations and measurements were performed twice by each observer, with an interval of 2 weeks between measurements to verify reproducibility of the technology.

First, we evaluated the mean values after four measurements, and second, we calculated Cohen’s kappa coefficient for inter- and intra-observer reliability. The hereby presented methodology was previously published in an analogue setting by the study group for the ACL [19].

### Statistical analysis

The reliability of photographic production and the marking of insertion site positions on our coordinate system was evaluated using the Cohen’s kappa coefficient for inter- and intra-observer reliability, for two observers. The kappa coefficient (*k*) is a parameter of intra-observer agreement for continuous outcomes, and ranges from 1 (perfect agreement) to 0 (no agreement). An a priori power analysis and sample size estimation were performed according to a previous investigation of the study group [18]. For statistical analyses, SPSS version 16.0 for Windows was used, and a *P*-value < 0.05 was considered statistically significant.

## Results

All tibial and femoral photographs were taken according to the described method, and were used to assess tibial and femoral attachment sites of the PCL using a coordinate system. The tibial and femoral coordinate points were measured with highly comparable inter-observer (*k* = 0.970) and intra-observer (*k* = 0.992) agreement, and may, therefore, be considered as highly reproducible measurements. Mean data after four measurements revealed a tibial insertion point T of 4.73/1.3 and a femoral insertion point F of 8.3/6.35, (Table 1).

**Table 1:** Mean values of tibial and femoral insertion points of the PCL after four measurements by two observers

	Tibial footprint	Femoral footprint
Observer 1 (first measurement)	4.8; 1.4	8.3; 6.6
Observer 2 (first measurement)	4.7; 1.2	8.4; 6.3
Observer 1 (second measurement)	4.7; 1.3	8.2; 6.2
Observer 2 (second measurement)	4.7; 1.3	8.3; 6.3
Mean	4.73; 1.3	8.3; 6.35

## Discussion

We observed that the tibial and femoral insertion site of the PCL was identified using our coordinate system, and we revealed a statistic significant intra- and inter-observer agreement (Cohen’s kappa coefficient). Given the fact that the PCL is one of the major passive stabilizers of the knee joint, restraining both posterior translation and rotation of the tibia, it appears obvious that PCL injuries inevitably lead to knee joint instability and secondary osteoarthritis [7, 12, 19, 23, 24, 28]. Since the PCL is believed to play a more expansive role in providing rotational stability than previously thought, it is important to assess internal and external rotation stability, in addition to posterior tibial translation when considering PCL injury [9]. The particular consequences of these findings may generate higher detection rates and increased attention for new PCL injuries, given the fact that PCL injuries have been historically underdiagnosed and estimated due to the high percentage of primary asymptomatic patients [11].

Surgical PCL reconstruction may be performed using different techniques, including single- and double-bundle reconstruction [14, 21, 23, 27]. However, some authors have described the superiority of double-bundle PCL reconstruction when compared to single-bundle techniques. The loading patterns of the two bundles, respectively, PCL biomechanics in general, seem to have not been completely investigated up to now [5, 22, 27, 29].

Traditionally believed to function independently during flexion (anterolateral bundle) and extension (posteromedial bundle) of the knee, the recent literature has described relationships between the two bundles as more synergistic and co-dominant, based on simultaneous elongation during a forward lunge [2, 10, 12, 23, 30]. However, due to high variability and data heterogeneity in these studies regarding load patterns of the PCL during normal daily activities, a definitive role for the ligament and its functional bundles remains controversial [20, 27].

Recent studies comparing anatomic single- versus double-bundle reconstruction techniques, suggest that the double-bundle technique closely approximates native knee kinematics, particularly beyond 90° flexion, respectively, also immediately after implantation [8, 18]. In contrast, Kim et al*.* determined no advantages of double-bundle over single-bundle PCL reconstruction, with respect to clinical outcomes or posterior knee stability [10]. Furthermore, single-bundle PCL reconstruction techniques have focused more on arthroscopic and radiographic reference points instead of the historical non-anatomic “isometric’’ reconstruction, with initial occurring joint over-constraint and progressive joint laxity described as complications [2, 3, 8, 10, 18, 20, 25, 27].

Our study has revealed that measurements of femoral and tibial insertion sites of the PCL are repeatable and reproducible, and therefore, accurate. Accordingly, this technique may represent a useful tool in preoperative planning for PCL reconstructions in the future. One possible application may be the preoperative determination of insertion sites using three dimensional-computed tomography reconstruction, with an overlaid coordinate system as presented here. The use of anatomical landmarks to create custom-made cutting blocks has already facilitated intraoperative handling in the setting of total knee arthroplasty [8].

This study has the following limitation: we assessed insertion sites of the PCL according to macroscopic findings, therefore, we did not distinguish between the insertion site of the anterolateral and the posteromedial bundle. However, we reiterate the following benefits: both PCL insertion sites were evaluated in a large number of human knees using standardized methods.

## Conclusions

Our study demonstrates that measurements of the femoral and tibial insertion sites of the PCL are repeatable, reproducible and highly accurate.

Our precise measurements of the PCL’s tibial and femoral footprint may facilitate intraoperative graft-placement in terms of determining the points of optimal repair for recreating the isometry of the PCL.
